# The role of liver resection in metastatic nephroblastoma: a systematic review and Meta-regression analysis

**DOI:** 10.1186/s12885-022-09182-3

**Published:** 2022-01-18

**Authors:** Juri Fuchs, Anastasia Murtha-Lemekhova, Markus Kessler, Patrick Günther, Katrin Hoffmann

**Affiliations:** 1 Department of General, Visceral and Transplantation Surgery, University Hospital Heidelberg, Heidelberg, Germany; 2 Department of General, Visceral and Transplantation Surgery, Division of Pediatric Surgery, University Hospital Heidelberg, Heidelberg, Germany; 3 Division of Liver surgery, Department of General, Visceral and Transplantation Surgery, University of Heidelberg, Im Neuenheimer Feld 420, 69120 Heidelberg, Germany

**Keywords:** Wilms’ tumor, Nephroblastoma, Liver metastasis, Pediatric liver surgery, Metastatic nephroblastoma, Hepatic metastasis, Stage IV nephroblastoma

## Abstract

**Background:**

The impact of hepatic resection for liver metastases (LM) on the survival of pediatric patients with Wilms’ tumor (WT) is unclear. So far, there is a lack of studies investigating the best suited treatment for patients with WTLM, and the role of liver resection has rarely been investigated. Thus, the development of evidence-based guidelines concerning indications of liver resection for WTLM remains difficult.

**Aim:**

To investigate the role of surgery in the therapy of WTLM. All available data on liver resections and subgroup outcomes of patients with WTLM are analyzed. Main research question is whether liver resection improves survival rates of patients with WTLM compared to non-surgical treatment.

**Methods:**

A systematic literature search of MEDLINE, Web of Science, and Central provided the basis for this PRISMA-compliant systematic review. For the main analysis (I), all studies reporting on surgical treatment of pediatric WTLM were included. To provide a representative overview of the general outcome of WTLM patients, in analysis II all studies with cohorts of at least five WTLM patients, regardless of the kind of treatment, were reviewed and analyzed. A Multiple meta-regression model was applied to investigate the impact liver resection on overall survival.

**Results:**

14 studies with reports of liver resection for WTLM were found (Analysis I). They included a total of 212 patients with WTLM, of which 93 underwent a liver resection. Most studies had a high risk of bias, and the quality was heterogenous. For the analysis II, eight studies with subgroups of at least five WTLM patients were found. The weighted mean overall survival (OS) of WTLM patients across the studies was 55% (SD 29). A higher rate of liver resection was a significant predictor of better OS in a multiple meta-regression model with 4 covariates (I2 29.43, coefficient 0.819, p = 0.038).

**Conclusions:**

This is the first systematic review on WTLM. Given a lack of suited studies that specifically investigated WTLM, ecological bias was high in our analyses. Generating evidence is complicated in rare pediatric conditions and this study must be viewed in this context. Meta-regression analyses suggest that liver resection may improve survival of patients with WTLM compared to non-surgical treatment. Especially patients with persisting disease after neoadjuvant chemotherapy but also patients with metachronous LM seem to benefit from resection. Complete resection of LM is vital to achieve higher OS. Studies that prospectively investigate the impact of surgery on survival compared to non-surgical treatment for WTLM are highly needed to further close the current evidence gap.

**Study Registration:**

PROSPERO 2021 CRD42021249763 https://www.crd.york.ac.uk/prospero/display_record.php?RecordID=249763.

**Supplementary Information:**

The online version contains supplementary material available at 10.1186/s12885-022-09182-3.

## Background

Wilms’ tumor (WT) is the fourth most common pediatric cancer and the most common malignant abdominal tumor in children [1]. 15% of all pediatric patients with nephroblastoma present with distant metastasis at diagnosis or with metastatic relapse [2]. About 20% of those suffer from liver metastasis (LM) [3–9]. Reported survival rates of these patients with LM differ substantially, also in recent studies, and range from 13 to 89% [3, 4, 7, 8, 10]. Especially patients with metachronous LM seem to have poorer survival compared to those with synchronous LM [9]. While complete remission of LM after sole chemotherapy has been reported [3, 4], the majority of WTLM patients were treated with liver resection (LR) in other studies [7]. Reviewing the literature, a lack of evidence concerning the role of LR for hepatic metastases of WT becomes apparent [11]. As a consequence, it is difficult for the important oncological study groups to draw up guidelines concerning the indication of LR for metastatic WT [12–15].

Aim of this systematic review is the comprehensive investigation of pediatric patients with WTLM, focusing on liver resection as treatment option. Outcomes of surgical treatment are compared with non-surgical therapy to evaluate whether LR improves survival rates. Potential harms or benefits of liver resection are investigated. All available data on LR for WTLM are analyzed to establish evidence-based recommendations for surgical treatment. Moreover, a data basis for future prospective trials on hepatic metastasectomy in pediatric WT is provided.

## Methods

### Structure, Search Strategy and Study Selection

This review was conducted in accordance with Preferred Reporting Items for Systematic Reviews and Meta-Analyses (PRISMA) guidelines [16, 17]. The study is based on a systematic methodology that has been specifically conceptualized for generating evidence in the field of rare liver afflictions. It is conducted within the framework of the *RELIVE* Initiative, a research consortium with the aim of establishing evidence-based therapies for rare liver diseases. Before starting the study selection, the methods were predefined, and the project was registered with the international Prospective Register of Systematic Reviews (PROSPERO 2021 CRD42021249763). Based on the evidence-based recommendations of the Study Center of the German Society of Surgery for the literature search strategy in surgical systematic reviews [41, 42], MEDLINE (via PubMed), Web of Science, and CENTRAL were searched applying a combination of the following medical subject heading (MeSH) and free text terms: nephroblastoma, wilm*, wilms tumor, metastases, metastasis, metastatic, stage IV, liver, hepatic, liver neoplasms. The full systematic search strategies are provided in the supplementary material 1. The last search was conducted on May 15th, 2021. In addition, reference lists of the relevant literature were screened for eligible studies. Except for review articles, all study types were eligible. Inclusion criteria were as follows: histologically proven diagnosis of nephroblastoma, liver metastases of nephroblastoma (synchronous and/or metachronous), pediatric patients (patient age < 18 years), and subgroup outcome of patients with WTLM reported. For the main analysis (Analysis I) of LR for WTLM, only studies that met the above-mentioned inclusion criteria and reported surgical treatment of WTLM were eligible. Cases with LR for direct hepatic invasion of right sided nephroblastoma were excluded. For a second analysis of the general outcome of patients with WTLM (Analysis II), all studies that included at least five patients with WTLM and reported subgroup outcomes of those patients were eligible, regardless of the applied treatment modality. Two reviewers (JF and AML) independently screened all abstracts that were found by the literature search for eligibility according to the defined criteria. Afterwards, the full texts of all eligible articles were assessed for inclusion by JF and AML independently.

### Data extraction

A form was set up that was used for standardized data extraction from all included studies. This form was validated with data extraction of the first five studies. The two reviewers (JF and AML) independently extracted the data according to this form. Collected information included authors names, year of publication, country, funding sources, number of patients reported, treatment groups, details on surgery, local treatment of metastases, outcomes, and follow-up.

### Risk of Bias Assessment and Certainty of Evidence

Concerning the main research question of this review, i.e., whether surgery for WTLM in children improves outcome compared to non-surgical treatment, no randomized-controlled trials were anticipated to be found. For observational studies, the validated Methodological Index for Non-randomized Studies (MINORS) tool was applied for risk of bias assessment (ROB) [18]. As case reports were included in this systematic review (SR), the tool by Murad et al. for ROB of case reports was used [19]. According to this method, the risk is rated in four domains: “Selection”, “Ascertainment”, “Causality” and “Reporting”. For each domain, the risk of bias is classified as either “low”, “moderate” or “high”. An overall judgement on the case report’s ROB is made after assessing each of the four domains individually.

The GRADE criteria were applied for rating the certainty of evidence and the strength of recommendations [20].

### Statistical Analyses

All statistical analyses were performed using R (version 3.6.2) [21]. For descriptive statistics, patient data was extracted and entered individually; means, medians and percentages with standard deviations (SD) were calculated. For an overview of the results across the different studies with larger cases series, (weighted) means or medians with (pooled) standard deviations (SD) were given for continuous data and numbers with percentages for categorial data. Depending on what data was given in the included publications, medians, means, and SD were calculated based on methods developed by Wan et al. [43]. As binary study endpoint, overall survival (OS) was defined as a patient being alive 2 years after the end of treatment. In some studies, event free survival (EFS) was reported, defined as time between end of first-line treatment and relapse or death of a patient. Univariate significance of distributions was tested with the chi-squared test at a level of significance of 5%.

None of the included studies had adequate intervention and control groups to allow for a classic meta-analysis (LR vs. no-LR). Instead, multiple meta-regression was applied to investigate the impact of the rate of liver resections performed in the study cohort on OS. All studies with more than 5 patients with WTLM and available outcome data were included. Heterogeneity among the studies was tested with Higgins & Thompson’s I^2^ [22]. A mixed-effects-model was applied for multiple meta-regression. Effect size was OS, as defined for our study. As the optimal local treatment of WTLM was the research question, the following predictors/covariates were predefined for the model:

1. Cohort size.
2. time of patient recruitment.
3. rate of patients with local radiotherapy.
4. rate of patients with liver resection..

All covariates were checked for multi-collinearity using a visualized correlation matrix. The Knapp-Hartung method was used to test the significance of predictors. P values < 0.05 were regarded as significant. The following packages were used for meta-regression analyses in R: *tidyverse, meta, metafor, and PerformanceAnalytics* [23–26].

## Results

### Literature Search and Study Selection

**Fig. 1:**
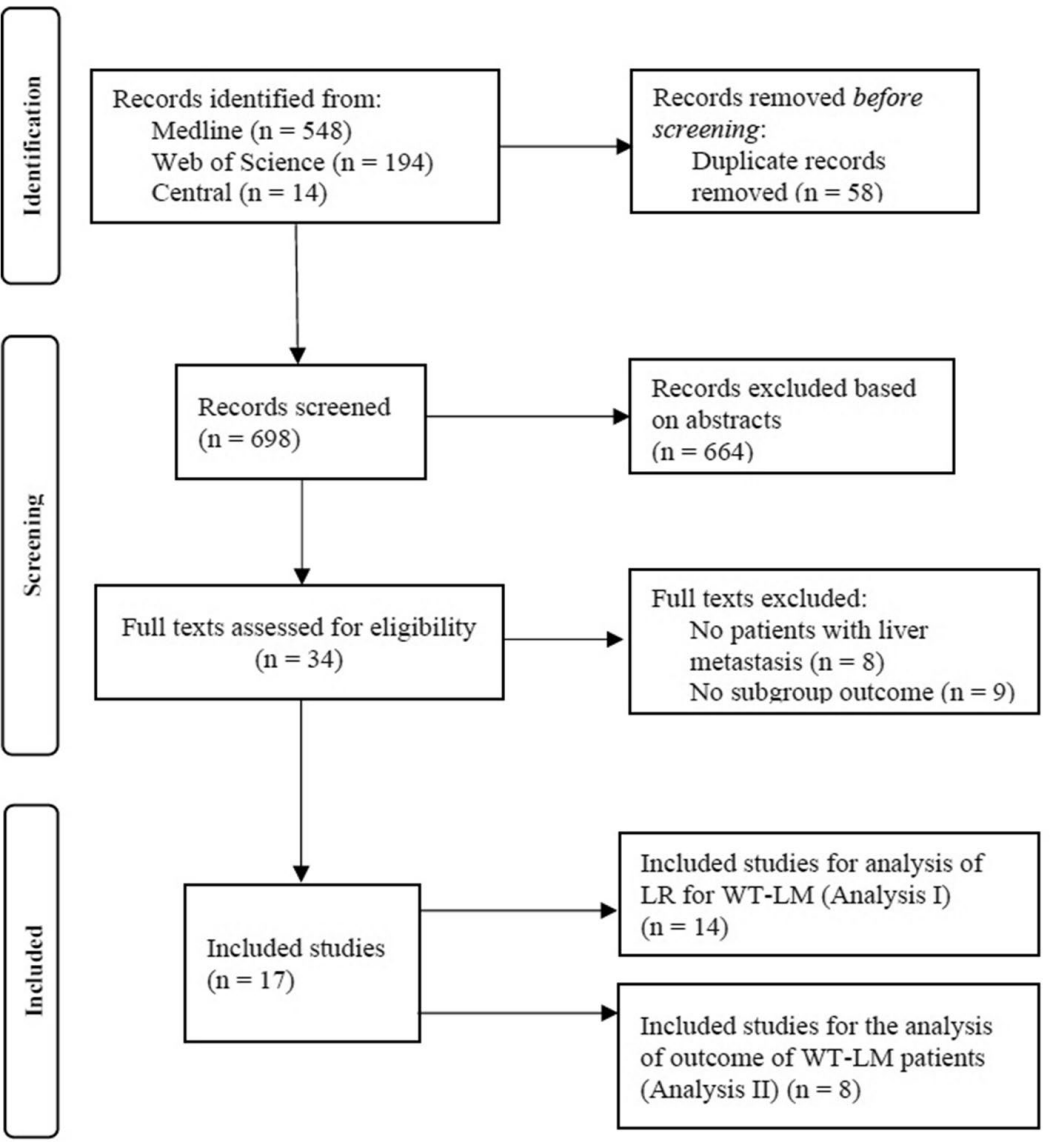
PRISMA flow chart of the study selection and inclusion process

The results of the literature search are depicted in the PRISMA flow chart (Fig. 1). 698 records were screened for eligibility. For the analysis I of LR for WTLM, 14 studies met inclusion criteria, reporting on a total of 212 patients with WTLM of which 93 underwent LR. For the analysis II that focused on the general outcome of WTLM patients with all kinds of local treatment, eight studies with a total of 254 patients with WTLM were included (among them five studies that were also included in analysis I).

In two studies, specific information on the outcome of patients with surgery for liver metastases was incomplete [4, 5]. Information was requested from the corresponding author in these cases, but the request was not met. As double reporting of patients could not be ruled in the two studies by Liné et al. [7, 27], the corresponding author was contacted. The request for clarification of which patients were reported in both studies was met. Thus, only three of the ten reported patients from the older and less comprehensive study [27] were included in this review and double reporting was avoided.

#### Overview of Included Studies, with Critical Appraisal and Risk of Bias Assessment

No randomized-controlled trials, propensity-score or matched-pair analyses were found.

##### Analysis I

There were three studies in which oncological trial registries were analyzed [3, 4, 7], one multi-center [6] (MC) and two single-center [5, 28] (SC) retrospective observational studies (ROS). The other eight studies were case reports or series [27, 29–35]. Five studies were comparative in that they included both, patients with surgical and patients with non-surgical treatment of WTLM [3–7]. However, only three of them adequately reported outcomes for these two groups separately to allow for an explorative comparison of interventions [3, 6, 7]. None of these five studies were conceptualized to specifically investigate the effect of surgery vs. no-surgery on the outcome of patients with WTLM. This implied that the two groups were not comparable regarding sample size, data on extent of disease and other possibly confounding factors. Selection bias was low, and the follow-up was long enough in the six ROS. The only study to report the extent of liver metastasis, type of resections and individual outcome for all patients, was the study by Liné et al. [7]. The case reports increased the risk of publication/reporting bias and tended to present favorable outcomes. Moreover, most of the case series or reports had a high selection bias. On the other hand, individual patient data were detailed in most case reports. However, data on the extent and location of LM were mostly insufficient throughout the included studies, which limited the comparison of the interventions.

##### Analysis II

Eight studies were found that reported subgroup outcome of a cohort >5 WTLM patients (irrespective of the kind of local treatment). Five of the eight included studies have also been included in analysis I and are discussed in this Sects. [3–7]. Another two ROS-SC studies were found that included pediatric patients with stage IV WT presenting with lung and/or liver metastasis [8, 10]. Both studies had no comparative design and local treatment for metastasis was either radiotherapy (RT) or not performed at all. The third study was a retrospective analysis of a large oncological trial registry [9]. This study compared a group of 236 patients with stage IV WT (synchronous lung and/or liver metastasis) to a group of 244 patients with relapsed WT and metachronous lung and/or liver metastasis. Local treatment of metastases was only briefly addressed in this study, and it was not reported how many patients received LR and/or RT as local therapy for LM. However, this study by Breslow et al. provided good quality regarding the prognostic difference of synchronous or metachronous WTLM. All three studies had adequate follow-up periods and a low selection bias. In summary, the quality of the existing studies on WTLM is low and most studies bear a moderate to high ROB. The studies were rather heterogenous, some with small caseload, and all had a retrospective design. A study designed to compare surgical vs. non-surgical treatment of LM has not been conducted yet. Results of the ROB are depicted in Tables 1 and 2.

**Table 1:** ROB of retrospective observational studies with MINORS [18]

MINORS Items	Breslow et al.		Su et al.	Fuchs et al.	Ehrlich et al.	Aronson et al.	Berger et al.	Liné et al.	Varan et al.	Jain et al.
Clearly stated aim		1	1	2	2	1	2	2	2	1
Inclusion of consecutive patients		2	2	2	2	2	2	2	2	2
Prospective collection of data		2	1	2	2	2	2	2	1	2
Endpoints appropriate to aim of study		1	2	2	1	1	2	2	2	2
Unbiased assessment of the study endpoint		1	0	1	1	1	1	2	0	0
Follow-up period appropriate to aim of study		2	1	2	2	2	2	2	2	2
Loss to follow up less than 5%		2	2	2	2	2	2	2	1	2
Prospective calculation of the study size		1	0	0	0	0	0	0	0	0
An adequate control group		1	-	1	1	0	0	1	1	-
Contemporary groups		2	-	2	2	2	2	2	2	-
Baseline equivalence of groups		1	-	1	0	0	0	0	1	-
Adequate statistical analyses		1	-	1	1	0	1	1	1	-
Total	17/24		9/16	18/24	16/24	13/24	16/24	18/24	15/24	10/16

**Table 2:** ROB of case reports/series according to Murad et al. [19]

Study	Selection	Ascertainment	Causality	Reporting	Overall ROB
Filler et al., 1969	high	moderate	high	moderate	high
Foster, 1978	moderate	moderate	high	moderate	moderate
Edwards et al., 1990	moderate	moderate	high	high	high
Rao et al., 1992	high	moderate	moderate	moderate	moderate
Goering et al., 2002	moderate	moderate	high	high	high
Patel et al., 2003	high	moderate	moderate	high	high
Dressler et al., 2010	high	moderate	high	high	high
Liné et al., 2014	low	moderate	moderate	moderate	moderate

#### Result of Analysis I: Liver Resection for Hepatic Metastases of Wilms’ Tumor

##### Patient characteristics

The 14 included studies reported on 212 pediatric patients with WTLM. Mean follow-up among the studies was 46 months (median 54, SD 31). Synchronous LM were found in 169 patients. 30 patients had a metachronous hepatic relapse. For 13 patients, the time of diagnosis of LM was not specified. Concurrent pulmonary metastases were present in 125 patients (59%). 67 patients had liver metastasis as only metastatic site (32%), and information on metastatic pattern was missing for 20 patients (9%). Table 3 gives an overview of the included studies of analysis I.

**Table 3:** Studies reporting on patients undergoing liver resection for metastatic Wilms’ tumor *Outcome only refers to 22 patients with resection of LM during primary tumor operation vs. 74 without primary resection of LM. No results for the subgroup of 25 patients with resection of LM available. Abbreviations: CR = case report; CS = Case series, EFS = event-free survival, M = metastasis; MC = multi-center, meta = metachronous liver metastases, OncReg = oncological study group registry, ROS = retrospective observational study, SC = single-center, sync = synchronous liver metastases, X = unknown

Authors	Study Type	Year of Publication	Recruitment period	Median age (SD)	Patients with WTLM	Concurrent pulmonary M	Outcome of WTLM Patients	Group A: LR	Outcome group A	Group B: no LR	Outcome group B
Filler et al.	CS	1969	1963-1968	4.4y	2 sync: 0 meta: 2	1/2 (50%)	Survival in 2/2 (100%)	**2**	Survival in 2/2 **(100%)**	0	-
Foster	CS	1978	1947-1978	-	15 Sync: X meta: 2+X	Unclear	Survival in 10/15 (67%)	**15**	Survival in 10/15 **(67%)**	0	-
Edwards et al.	CS	1990	1978-1987	10y	1 sync: 0 meta: 1	0/1	Survival in 1/1	**1**	Survival in 1/1	0	-
Rao et al.	CR	1992	-	8y	1 sync: 0 meta: 1	0/1	Survival in 1/1	**1**	Survival in 1/1	0	-
Goering et al.	CR	2002	-	-	1 sync: 0 meta: 1	Unclear	Survival in 1/1	**1**	Survival in 1/1	0	-
Patel et al.	CR	2003	-	0.8y	1 sync: 0 meta: 1	0/1	Survival in 1/1	**1**	Survival in 1/1	0	-
Su et al.	ROS-SC	2007	1988 - 2005	6.2y (7.1)	3 sync: 1 meta: 2	1/3 (33%)	Survival in 0/3 (0%)	**3**	Survival in 0/3 **(0%)**	0	-
Fuchs et al.	ROS-OncReg	2008	1994-2004	6.5y (8.6)	45 sync: 29 meta: 16	28/45 (62%)	Survival in 24/45 (53%)	**21**	Survival in 12/21 **(57%)**	24	Survival in 12/24 (50%)
Ehrlich et al.	ROS-OncReg	2009	1986-2002	-	96 sync: 96 meta: 0	62/96 (65%)	-	**25**	5-year EFS 86%*	71	5-year EFS 68%*
Dressler et al.	CR	2010	-	9y	1 sync: 0 meta: 1	1/1	Survival in 1/1	**1**	Survival in 1/1	0	-
Aronson et al.	ROS-SC	2012	2002-2010	3.8y (3.2)	19 sync: 19 meta: 0	15/19 (79%)	5-year OS 47%	**3**	unknown	16	unknown
Berger et al.	ROS-MC	2013	1994-2011	5.4y (4.2)	6 sync: 6 meta: 0	3/6 (50%)	Survival in 3/6 (50%)	**2**	Survival in 2/2 **(100%)**	4	Survival in 1/4 (25%)
Liné et al.	CS	2014	1994-2001	1.9y (2.6)	3 sync: 0 meta: 3	Unclear	Survival in 2/3 (67%)	**3**	Survival 2/3 **(67%)**	0	-
Liné et al.	ROS-OncReg	2020	2002-2012	6y (2.6)	18 sync: 18 meta: 0	14/18 (78%)	Survival in 16/18 (89%)	**14**	Survival in 12/14 **(86%)**	4	Survival in 4/4 (100%)

##### Liver resection for WTLM

In 93 of the 212 patients (44%), LR was performed. 29 (31%) atypical or wedge resections and 24 (26%) anatomical major LRs were reported. In 40 cases (43%), data on the type of surgical procedure were missing. Complete tumor resection was confirmed in 36 operations (39%). Incomplete resection was reported in 16 patients (17%). For 41 patients, there were no adequate data on the resection status (44%). Hepatic re-resections were performed in 16 children (17%). (See Table 4)

**Table 4:** Details on surgical procedures

All patients with LR for WTLM n = 93	
Atypical LR	29 (31%)
Major anatomic LR	24 (26%)
Not specified	40 (43%)
Complete resection	36 (39%)
Incomplete resection	16 (17%)
Resection status unknown	41 (44%)
Postoperative deaths	2 (2%)
Major complications^a^ (reported)	4 (4%)

^a^ Clavien-Dindo grade III or higher

**Fig. 2:**
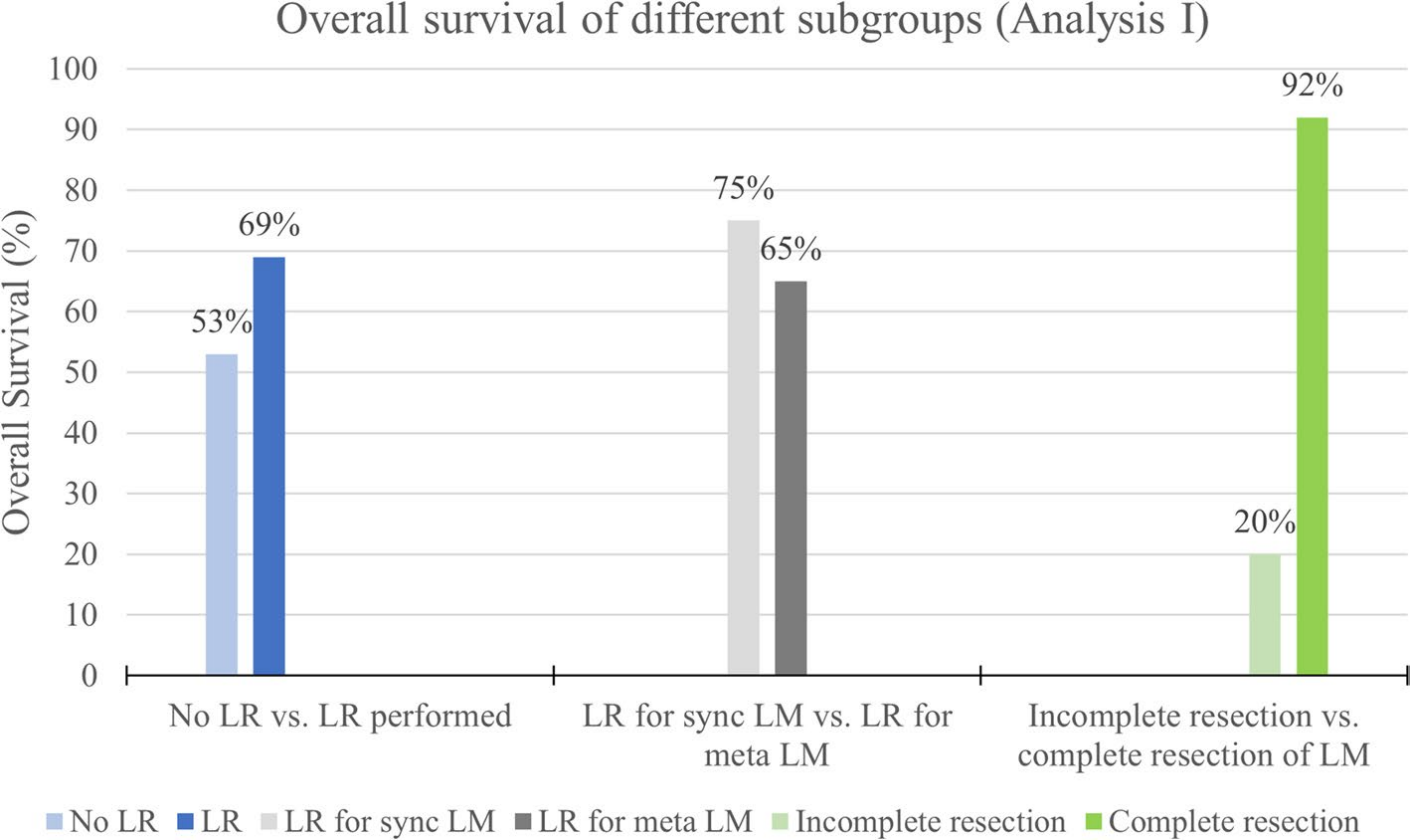
Overall survival rates of different subgroups in Analysis I (OS rates refer to subgroups of patients with available outcome data). LR = Liver resection, Meta = metachronous liver metastasis, Sync = synchronous liver metastasis

Among the 93 patients with LR, 60 had synchronous LM (65%), 20 had metachronous LM (22%), and for 13 patients (14%), the time of diagnosis of LM was not reported. Data on overall survival was available for 65 of all 93 patients who underwent LR. For those patients, OS was 69% and 20 deaths occurred in the reported follow-up period (31%). In the subgroup of patients with synchronous LM, OS was 75%. In the subgroup of patients with metachronous LM, OS was 65%. For the 36 patients with confirmed tumor free resection margins, OS was 92% (33 patients survived, three deaths in the reported follow-up period). (See Table 5; Fig. 2.)

**Table 5:** Outcome of LR in different subgroups

	All patients with surgery for WTLM n = 93			Synchronous LM n = 60	Metachronous LM n = 20	Time of LM-diagnosis not specified n = 13	Complete resection of LM confirmed n = 36
OS		45 (48%)		24 (40%)	13 (65%)	8 (62%)	33 (92%)
Disease-related death		20 (22%)		8 (13%)	7 (35%)	5 (38%)	3 (8%)
Insufficient outcome data		28 (30%)		28 (47%)	-	-	-

##### Non-surgical treatment of WTLM

119 patients of the 212 did not undergo LR. For most of them, no subgroup outcome was reported. For 32 patients without LR, data on OS were available. OS was 53% (17 patients), 47% (15 patients) died of progressive disease.

##### Chemotherapy and timing of Liver resection

Information on applied chemotherapeutic regimens and the timing of LR was available for 70 of the 93 patients with LR. 48 patients (69%) received neoadjuvant chemotherapy before LR was performed. In 27 of those, LR was performed together with resection of the primary tumor. In 21 patients, LR was performed as a separate operation after neoadjuvant chemotherapy and resection of the primary tumor. In 22 patients (31%), upfront surgery with resection of the primary tumor and concurrent LR was performed before starting any kind of chemotherapy, followed by adjuvant chemotherapy in all 22 cases. OS of these different subgroups was not available based on the data of the included studies.

##### Comparative studies

Five studies included both, patients with LR for WTLM and patients without surgery. Only the study by Liné et al. presented details on the treatment algorithm [7], that led to the decision of performing or not performing LR. The other studies were lacking those data. The study with the largest cohort of patients with WTLM (only synchronous) did not report the outcome of the LR subgroup [4]. The only subgroup analyses performed in this NWTS study by Ehrlich et al. was a comparison of Event free survival (EFS) between 22 patients with LR for LM during the primary tumor operation versus 75 patients without primary LR. The group with LR had better EFS (5-year EFS 86%) compared to the group without (5-year FS 68%), but this difference was not significant. Another 4 patients received LR later during therapy, making for a total of 25 patients who underwent LR in this cohort. However, no outcome was reported for this subgroup of 25 patients with LR. Fuchs et al. analyzed 45 patients with WTLM treated within the Société Internationale d’Oncologie Pédiatrique/International Society of Paediatric Oncology (SIOP)/ Gesellschaft für pädiatrische Onkologie und Hämatologie (GPOH) studies [3]. 29 had synchronous and 16 metachronous LM. In total, 21 patients underwent LR and 24 were treated without surgery for LM. OS was 57% in the group with surgery (12/21) and in 50% in those without surgery for LM (12/24), the difference being not significant (OR 1.3, 95%-CI 0.3-5.1, *p* = 0.631). OS was 100% in the 11 patients with complete resection of LM. In the study by Berger et al., 2 of the 6 patients with LM underwent LR. OS of those patients was 100%, while it was 25% for patients without LR [6]. Liné et al. presented 18 patients with synchronous WTLM of whom 14 received LR [7]. OS was 86% for these patients with LR (12 patients). The analysis of Aronson et al. did not include subgroup outcomes of the 3 patients with LR and the 13 with non-surgical treatment of LM [5]. Overall, the resection rate for WTLM varied from 16 to 79% (median 33.3%, SD 21) across the five studies.

##### Histology

High-risk histology (HR) was defined as blastemal predominance or diffuse anaplasia. Other subtypes were defined as non-HR (including low-risk and intermediate-risk). In five studies, information on histologic subtypes of the patients’ primary tumor and/or liver metastases were given [3–7]. Thus, information on histology was available for 184 of 212 WTLM patients (with or without LR). In the largest study on WTLM patients, children with HR were excluded from the analysis [4]. In total, 24 patients (13%) patients had HR, and 171 non-HR (93%). Diffuse Anaplasia was found in 11 patients (6%). Separate reports on the outcome of the histologic subgroups were only partly available. In the study by Fuchs et al., OS among the 9 patients with HR was 22%. Liné et al. analyzed 4 patients with HR: two with blastemal predominance and two with diffuse anaplasia. All four underwent LR. Both patients with blastemal subtype were long-term survivors, while the two patients with diffuse anaplasia both suffered from recurrence and died.

#### Results of Analysis II: Outcome of Patients with Liver Metastasis of Wilms’ Tumor – Irrespective of the Kind of Local Treatment

**Fig. 3:**
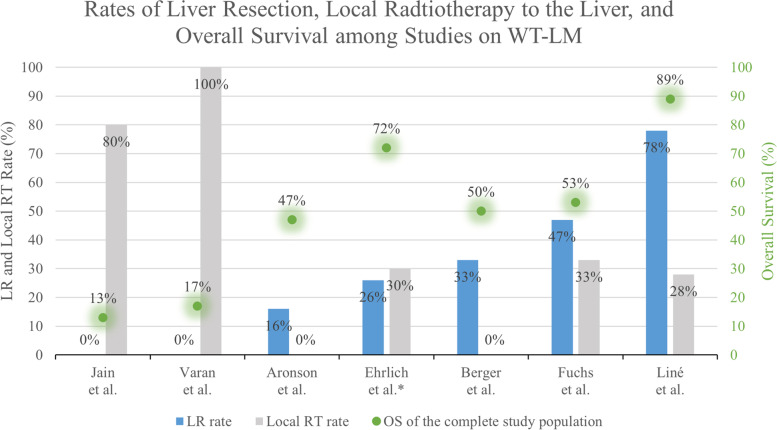
Rates of liver resection, radiotherapy, and overall survival of the study cohort among the different included studies of patients with liver metastasis of wilms’ tumor. *No patients with high-risk histology included in the study. LR = liver resection, RT = radiotherapy, OS = overall survival

**Fig. 4:**
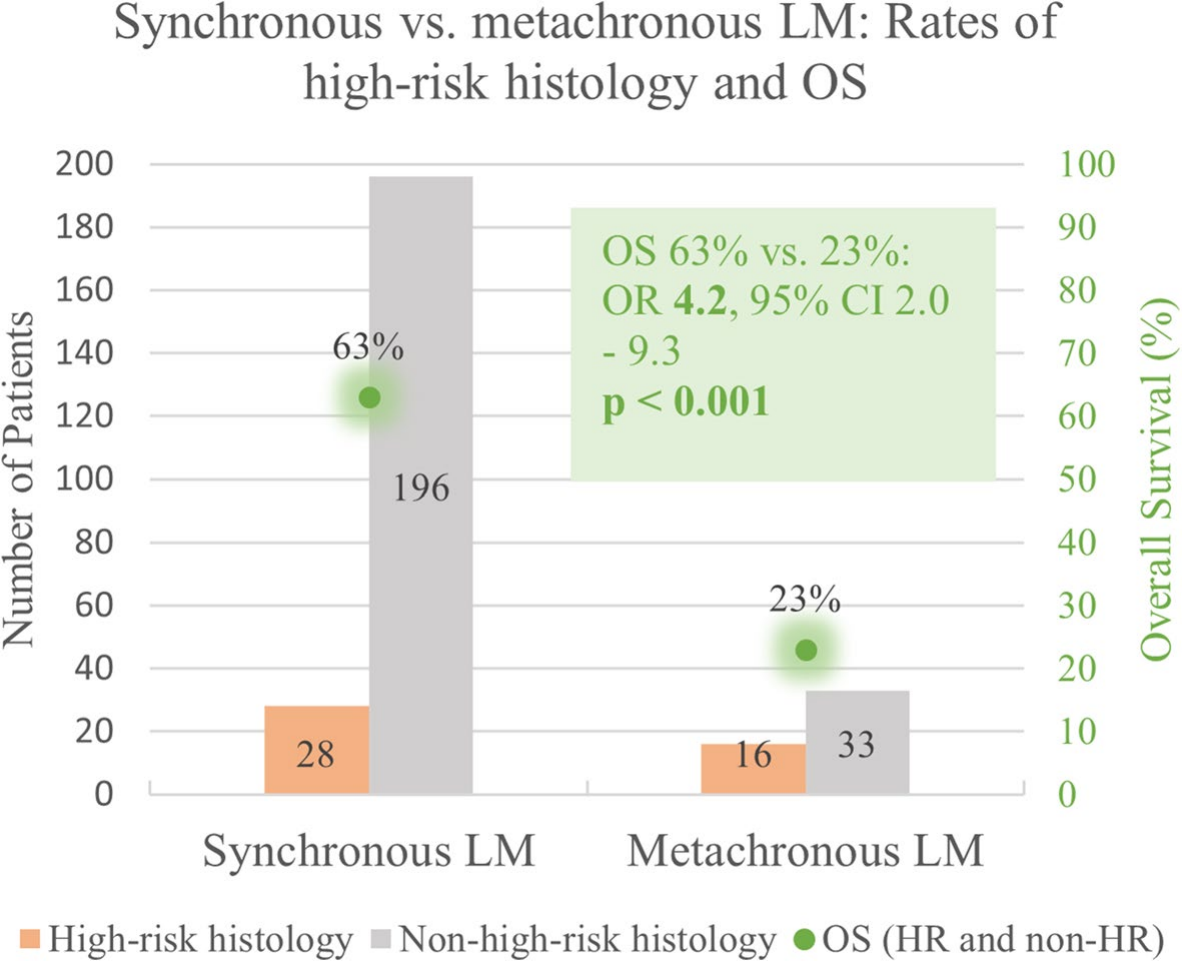
Comparison of synchronous vs. metachronous liver metastases with percentage of patients with high-risk histology and overall survival of the two groups. LM = liver metastasis, HR = high-risk histology

Studies with large cohorts of patients with metastatic WT that included at least five cases with WTLM and reported subgroup outcome were eligible for this analysis. All kinds of treatments for WTLM were included, meaning that patients either underwent no local therapy for LM (chemotherapy only), received LR plus chemotherapy, RT plus chemotherapy, or a combination of LR, RT and chemotherapy. Eight studies were found that reported the subgroup outcome of patients with WTLM (four of them have already been included in analysis I). A total of 273 WTLM patients were analyzed, of which 224 had synchronous, and 49 metachronous LM. 44 patients (16%) had HR. Patients with metachronous LM had significantly more often HR compared to those with synchronous LM (33% vs. 13%, OR 3.4, 95% CI 1.6 – 7.4, p < 0.001). The local treatment regimens differed among the studies. Radiotherapy (RT) was the only applied local treatment of LM in two studies [8, 10]. One trial predominantly applied local RT and only few received LR [9]. In one cohort, RT and LR were applied with a similar rate [4]. A higher rate of LR and lower administration of RT was used in another two studies [3, 7]. In two studies, the only applied local treatment for LM was surgery [5, 6]. The outcome varied among the studies, with OS ranging from 13 to 89%. The weighted mean OS of all WTLM patients across the seven studies was 55% (SD 29). For patients with synchronous LM, OS was significantly higher than for those with metachronous LM (63% vs. 22.5%, OR 4.2, 95%-CI 2.0-9.3, *p* < 0.001). Table 6 gives an overview of the included studies in analysis II. (See Figs. 3 and 4)

**Table 6:** Studies with cohorts of Stage IV/metastatic WT that reported subgroup outcome of patients with LM (only studies reporting > 5 patients with LM)

Authors	Study Type	Year of Publication	Recruitment period	Study population	Number of patients with LM	WTLM with high-risk histology		Local treatment of LM		Outcomes
Breslow et al.	ROS-OncReg	1986	1969-1983	480 patients with Stage IV or metastatic WT Sync: 236 with lung and/or liver M Meta: 244 with relapse in lung and/or liver	**69** Sync: 36 Meta: 33		15 (22%) Sync: 4 (11%) Meta: 11 (33%)	RT and/or surgery (numbers unknown)		LM (all): OS **39% (SD 5)** Sync LM: OS **64% (SD 8)** All sync patients: OS 67% (SD 3) Meta LM: OS **12% (SD 6)** All meta patients: OS 40% (SD 3)
Varan et al.	ROS-SC	2005	1971-2002	57 patients with Stage IV WT (sync only, different metastatic sites)	**12** (sync only)		3 (25%)	RT only		LM (all): OS **17% (SD 11)** All Stage IV patients: OS 37% (SD 6) M lung only: OS 50% (SD 7)
Fuchs et al.	ROS-OncReg	2008	1994-2004	45 patients with WTLM Sync: 29 Stage IV Meta: 26 relapsed	**45** Sync: 29 Meta: 16		9 (20%) Sync: 4 (14%) Meta: 5 (31%)	Surgery: 21 RT: 23		LM (all): OS **53% (SD 7)** Sync: OS 59% (SD 9) Meta: OS 44% (SD 12)
Ehrlich et al.	ROS-OncReg	2009	1986-2002	96 patients with WTLM (sync only)	**96** (sync only)		0	Surgery: 25 RT: 26		LM (all): OS **72% (SD 6)**
Berger et al.	ROS-MC	2013	1994-2011	31 patients with stage IV WT (sync only, different metastatic sites)	**6** (sync only)		3 (50%)	Surgery: 2 RT: 0		LM (all): OS **50% (SD 20)** All stage IV patients: OS 82% (SD 7)
Aronson et al.	ROS-SC	2016	2002-2010	45 patients with stage IV WT (sync only)	**19** (sync only)		9 (47%)	Surgery: 3 RT: 0		LM (all): OS **47% (SD 11)** All stage IV patients: OS 59% (SD 7)
Jain et al.	ROS-SC	2020	2000-2012	36 patients with Stage IV WT, lung and/or liver M (sync only)	**8** (sync only)		1 (13%)	RT only		LM (all): OS **13% (SD 12)** All Stage IV patients: OS 50% (SD 8)
Liné et al.	ROS-OncReg	2020	2002-2012	18 patients with WTLM (sync only)	**18** (sync only)		4 (22%)	Surgery:16 RT: 6		LM (all): OS **89**% (SD 7)
Pooled data				808	**273** Sync: 224 Meta: 49		**44** Sync: 28 (13%, SD 5) Meta: 16 (33%, SD 7) → p < 0.001		Weighted mean OS (LM all): **55%** (SD 24) LM Sync: **63%** (SD 27) LM Meta: **23%** (SD 9) → p < 0.001	

Abbreviations: LM = Liver metastasis, M = metastasis, MC = multi-center, meta = metachronous liver metastases, OncReg = oncological study group registry, OS = overall survival, ROS = retrospective observational study, SC = single-center, sync = synchronous liver metastases

#### Multiple Meta-Regression Analysis

The 7 studies [3–8, 10, 36] with subgroups of WTLM patients < 5 were included in a multiple meta-regression model for OS with 4 covariates, as explained in the 2 section. I^2^ (residual heterogeneity) was 29% (See Table 7). 94% of the heterogeneity of the effect sizes among the trials (i.e., differences in OS), were explained by the multiple meta-regression model (R^2^ = 94.31%). Liver resection rate was the only significant predictor of OS, meaning that higher rates of liver resection were associated with higher OS (coefficient 0.819, p = 0.038). See Table 7.

**Table 7:** Results of multiple meta-regression

Studies analyzed n = 7		
Effect size: Overall survival		
I^2^ (residual heterogeneity): 29.43% R^2^ (amount of heterogeneity accounted for): 94.31%		
Predictor	Coefficient estimate	*p* value
Study cohort size	0.002	0.308
Recruitment period	0.002	0.746
**Rate of liver resections**	**0.819**	**0.038**
Rate of local radiotherapy	-0.288	0.276

## Discussion

In this first systematic review on the outcome and therapy of pediatric patients with liver metastases of nephroblastoma, a total of 301 patients were analyzed. Against the background of an alarming lack of evidence concerning the role of surgery and the best suited local treatment for WTLM, this study is an initial step towards an evidence-based therapy and can function as a starting point for vitally needed prospective studies in the future. Albeit bearing a high risk of ecological bias, a multiple meta-regression model showed that higher rates of liver resection were significantly associated with higher survival rates of pediatric patients with liver metastases of Wilms’ tumor, which may point to a benefit of liver resection for WTLM patients.

### Overview on the Role of Liver Resection for WTLM

This review reveals that no study has been conducted so far, that specifically investigated the best suited local treatment regimen for WTLM. The results of our meta-regression model suggest that liver resection achieves favorable outcomes compared to non-surgical treatment of WTLM. Moreover, we showed that the rate of LR in WTLM patients differs substantially across different studies, including large trials of the important pediatric oncological study groups [3, 4, 7]. While 78% of French patients with WTLM in the SIOP2001 study [7] underwent LR, only 26% were treated with surgery as local treatment for LM in the National Wilms’ Tumor Study Group (NWTS) trials 4 and 5 in the US [4]. This discrepancy can be explained by the lack of evidence on the best suited local treatment for WTLM, resulting in ambiguous and heterogenous recommendations. Albeit vague regarding the extent of surgery and possible contraindications, the European-based SIOP guidelines recommend the surgical resection of persisting WT metastases after neoadjuvant treatment [7]. In case of complete resection of metastases, local RT to the metastatic site in not generally advised [7]. In the guidelines on the local treatment of WT metastases by the US-American Children’s Oncology Group (COG), surgical resection is not explicitly recommended, while most patients receive RT to the site of metastasis [4, 37].

### Outcome of WTLM patients

The outcome of pediatric WT has dramatically improved over the last decades with OS above 90% [11]. However, patients suffering from metastasis have an increased risk of therapeutic failure [11]. We showed that the liver as site of metastatic relapse is associated with poorer survival than the lung [3, 9, 38]. In the present analysis, the survival rate of WTLM patients in the included studies ranged from 13 to 89%, and the weighted mean was 55%. Moreover, our results clearly show that patients with metachronous LM have a poorer prognosis than those with synchronous (23% vs. 63%, p < 0.001). Our analysis points out that WT patients with complete surgical resection of LM have an excellent prognosis (OS 92%).

### Surgery for Synchronous Liver Metastasis

The weighted mean survival of patients with synchronous WTLM among the included studies was 63% (SD 27). Separately analyzing the patients with synchronous LM who were treated with LR (and outcome data available), 75% survived. Results of the few existing studies on LR for WTLM suggest better outcome of patients undergoing surgery for hepatic metastasis compared to those without LR. For example, children with WTLM treated in the NWTS-4 and -5 trials achieved higher EFS when receiving LR during initial primary tumor surgery compared to those who did not (86% vs. 68%) [4]. Liné et al. reported OS of 86% in 14 patients with LR, of whom only five (31%) received RT to the liver. The results of both studies indicate a favorable role of LR in synchronous WTLM. In the two studies with RT as sole local treatment for synchronous WTLM, survival was only 13% and 17%, respectively [8, 10]. However, it should be noted that these studies present populations with differing characteristics, and the dramatic discrepancies in the outcome can be caused by additional factors. The excellent results in the study of Liné et al., where all patients initially received neoadjuvant chemotherapy according to SIOP guidelines and a high percentage of patients underwent LR (78%), prompt the conclusion that this treatment algorithm can be recommended. Moreover, surgical metastasectomy might allow for a reduction of doses or omission of RT to the liver and thus avoid proven acute and long-term toxicity of radiation in the liver [39, 40].

### Surgery for Metachronous Liver Metastasis

Weighted mean survival of patients with metachronous WTLM among the included studies was 23%. Looking only at the patients with metachronous LM who were treated with LR, 65% survived. This suggests that those patients benefit from surgical treatment of metastatic relapse in the liver. The impact of a possible selection bias could not be fully clarified, given the quality of the existing studies. Patients treated with surgery for LM might have had favorable characteristics, that led to a better outcome. However, considering the poor outcome of patients with metachronous WTLM shown in our study, treatment concepts are highly needed and LR plays an important role in these cases.

### Safety of Liver Resection in Children

Reported postoperative mortality was 2% and major surgical complications were reported in 4% across all included cases (two cases of posthepatectomy hemorrhage, one case each of intraoperative hemorrhage and postoperative sepsis). However, most studies did not report on postoperative complications with standardized and reliable methods, which is a major limitation concerning the evaluation of potential risks of liver resection for WTLM. Nevertheless, the low rate of surgical and postoperative complications reported in those studies with adequate reporting of postoperative outcome suggests that patient safety in liver resection in pediatric WTLM patients can be regarded as high. Looking at the literature on morbidity and mortality in pediatric liver resection in general, a lack of evidence becomes apparent [44, 45]. In particular, standardized and thus comparable reporting of postoperative outcome and complications has not been widely established yet [45]. The rates of postoperative complications in studies on pediatric liver resection range from 10 to 69%, with mortality rates between 0 and 5% [45–48]. For hepatic metastasectomy in children specifically, only one study has been published with a reported morbidity of 13% (one wound infection and one bile leakage) [28]. Concerning late sequelae of liver resection in pediatric patients, the follow up period of the included studies in our review was too short to gain insights. In large cohort studies of long-term effects of cancer treatment in pediatric oncological patients, abdominal surgery, including liver surgery, was not an independent predictor of liver-specific nor gastrointestinal morbidity in adult life [49, 50]. Overall, initial evidence generated by our study suggests that liver resection of WTLM in children has a moderate risk profile. Future studies should report on postoperative outcomes using standardized methods to allow for a better comparison of results.

### Patient Selection for Liver Resection of Wilms’ Tumor Metastases

Our study results show that presence of concurrent metastasis at other sites except for the liver, represent no contraindication for resection of WTLM. In fact, most pediatric patients with WTLM also have pulmonary metastases [3, 4, 6, 7]. For patients with histology of blastemal predominance, and especially for all those with favorable histology (non-high-risk), surgical treatment for WTLM can be recommended even in cases of disseminated LM, as shown by the results in the study of Liné et al. [7]. Likewise, Fuchs et al. showed that the prognosis is excellent when complete resection of LM is achieved [3]. OS was 100% for patients with complete surgical resection of WTLM in their study [3]. Cohorts in which none of the WTLM patients received LR, showed poor survival [8, 10] and there was a trend towards higher OS in cohorts with higher LR rates. This finding further strengthens the positive impact of LR, for at least a subset of WTLM patients. However, initial evidence suggests that in patients with diffuse anaplasia, indication for extensive surgical treatment in cases of disseminated disease should be made reluctantly. The benefit of metastasectomy for the prognosis of these patients remains questionable according to our findings. Given the unfavorable prognosis for these patients, studies are highly needed and the role surgical metastasectomy should be further investigated. Another finding of this analysis is that R0 resection of LM results in excellent survival rates for WTLM patients. This emphasizes that careful preoperative operation planning is mandatory in order to avoid residual disease or positive resection margins after LR. It might include combinations of anatomic and atypical resection, innovative strategies such as combinations of surgical resection with radiofrequency ablation [35], or, if necessary, extensive resections.

### Limitations and Strengths of the Study

Main limitation of this systematic review is the lack of comparative studies investigating the best suited local treatment for WTLM. Direct comparison of interventions was restrained by the limited quality and a high ROB of most of the included studies. As a result, ecological bias of our analyses is high, and our results must be evaluated in this context. Major strength of the study is the comprehensive analysis of all published data on LR for pediatric WTLM and the summary of all reported outcomes of subgroups larger than five patients with WTLM. Moreover, a multiple meta-regression model showed that liver resection improved outcome compared to non-surgical treatment. Thus, our results might function as basis and reference point for all future studies investigating prognostic factors and improvements in the therapy of pediatric patients with WTLM. We provide a sound data basis for the development of future prospective trials and evidence-based guidelines.

### Certainty of Evidence and Strength of Recommendations

Only observational trials have been published that investigated the outcome of patients with WTLM. Thus, the certainty of evidence of the current systematic review is low. The strength of all recommendations derived from this systematic review is conditional. However, our study provides the highest level of evidence available on the role of LR, outcomes and local treatment concepts for WTLM hitherto reported.

## Conclusions

This is the first systematic review on WTLM ever conducted. Generating evidence is complicated in rare pediatric conditions and this study must be viewed in this context. Nevertheless, this systematic summary and analysis of all available data on WTLM produced several valuable insights. Our study reveals that the question of the best-suited treatment of liver metastasis of nephroblastoma in children has been neglected so far and there is a lack of studies that specifically investigated this condition. While some studies with adequate design and acceptable risk of bias were found, the quality of the included studies differed substantially. As a result, most of the conducted analyses bear a risk of bias, such as ecological bias. However, meta-regression analyses suggest that liver resection may improve survival of patients with WTLM compared to non-surgical treatment. Moreover, our results point to a low morbidity of liver resection for WTLM. It seems vital to achieve a complete resection of LM to reach better survival rates for the affected patients. These results can function as an important starting point for further research and as initial reference for clinical recommendations. Future studies, ideally with a randomized design, that prospectively investigate the impact of surgery on survival compared to non-surgical treatment for WTLM are highly needed to further close the current evidence gap.

### Key Points

- According to the available data, surgical therapy for liver metastasis of Wilms’ tumor may achieve higher survival rates than non-surgical treatment.
- Liver resection for metastatic Wilms’ tumor in pediatric patients is associated with a moderate risk of postoperative complications.
- Prognosis of WTLM is clearly improved when complete surgical resection is achieved.
- Metachronous liver metastasis of nephroblastoma is associated with poor survival.
- Prospective studies, ideally with randomization, investigating the best-suited local treatment regimen for liver metastases of Wilms’ tumor are vitally needed.

## Supplementary Information

**Additional file 1.**

## Data Availability

Publicly available datasets were analyzed in this study. The full search strategy used for this study is provided.

## Abbreviations

COG: Children’s Oncology Group
EFS: Event free survival
HR: High-risk histology
LM: Liver metastasis
LR: Liver resection
M: Metastasis
Meta: Metachronous metastasis
NWTS: National Wilms’ Tumor Study
OS: Overall survival
ROB: Risk of bias
RT: Radiotherapy
SIOP: Société Internationale d’Oncologie Pédiatrique
SR: Systematic review
Sync: Synchronous metastasi
WT: Wilms’ Tumor/Nephroblastoma
WTLM: Liver metastasis of Wilms’ tumor
