# Supplementary material for: The role of liver resection in metastatic nephroblastoma: a systematic review and Meta-regression analysis
Source: BMC Cancer. 2022 Jan 18;22:76. doi: 10.1186/s12885-022-09182-3 (PMC8764777; doi:10.1186/s12885-022-09182-3)
Supplement: Supplementary file 1 — Additional file 1. [file 12885_2022_9182_MOESM1_ESM.docx]

**Search strategy for “The Role of Liver Resection for Metastatic Nephroblastoma: A Systematic Review and Pooled Data Analysis”:**

Medline (via Pubmed):

((liver[tiab] or hepatic[tiab]) AND (metastatic[tiab] OR metastasis[tiab] OR metastases[tiab] OR Stage IV[tiab] OR Stage 4[tiab]) AND (nephroblastoma[tiab] OR wilm*[tiab])) OR (Liver neoplasms[MeSH] AND Wilms Tumor[MeSH])

Web of science:

TS = ((nephroblastoma OR wilm*) AND (liver OR hepatic) AND (metastasis OR metastases OR metastatic OR stage IV) )

Central:

(nephroblastoma OR wilm*) AND (Liver OR hepatic) AND (metastatic OR metastasis OR metastases OR Stage IV)
